# Some Ward Sisters and Staff Nurses Protest

**Published:** 1916-08-26

**Authors:** 


### THE KING GEORGE HOSPITAL.

### Some Ward Sisters and Staff Nurses Protest.

With reference to an article entitled "*Not* 'Good Enough for the War,'" on page 473 of our last issue, we have received a statement signed by thirty-four ward sisters and forty staff nurses of King George Hospital, which we print below. In the course of this article we pointed out that it was mischievous and wrong in the administrative sense to disregard the interests of an enormous staff of workers and of an infinite and ever-changing number of sick and wounded warriors under treatment in a hospital.

The staff of King George Hospital in February 1915 was stated to consist of three principal sisters, ten senior sisters, thirty-seven ward and theatre sisters, 228 staff nurses, and eighty V.A.D. members, in addition to a certain number of male orderlies. The hospital contained 1,650 beds. It will be observed that the manifesto printed below is representative so far as the ward sisters are concerned, whilst less than one in five of the staff nurses have appended their names. This fact should be noted, as it seems to demonstrate the independence of the staff nurses and their hope for the institution of changes which will make their lot happier and provide that their sleeping accommodation shall be so arranged as to give to each nurse adequate privacy and comfort.

The wording of the manifesto practically admits the existence of the conditions at King George Hospital, which we condemned on the ground that no hospital has the right to gamble in the health of its nursing staff by providing them with sleeping accommodation which is far below the standard of modern hospital construction. It upholds the matron who was only an incident in the point of view insisted upon in our article last week, and justifies the position by declaring that each of the signatories entered the service of King George Hospital with a full knowledge of the conditions under which they would have to live. We hold that every hospital, both military and civil, should have a standard of efficiency equally applicable to the accommodation provided and to the demands made upon each worker for loyal, self-denying and arduous service. Our position is that this standard is non-existent in regard to the accommodation provided at this hospital, whilst its nursing staff have admittedly maintained this standard so far as their work and service are concerned. This being so, the protest made by some sisters and nurses errs, if it errs at all, on the side of extreme generosity, whilst it confirms and indeed admits the truth of our main contention, and makes it plain that the conditions under which they voluntarily took office were such as no other hospital conducted on the highest principles could have suggested for the acceptance of the members of its staff.

Of all employers the managers of a hospital have the greatest responsibility in regard to the presence of the best hygienic conditions throughout their establishment, and the provision of the best possible means for maintaining the health and comfort of every worker connected with it. Cubicles under the best of circumstances are not suitable as sleeping accommodation for educated women engaged in nursing the sick in a big hospital in the present day. The worst form of cubicle is the large dormitory divided by curtains into floor spaces only, each containing a bed—the form to be seen at the King George Hospital—for it is inimical to comfort, devoid of privacy and adequate ventilation, and tends to keep the occupants awake and to make them peculiarly susceptible to every sound and movement. The primary fault was to accept buildings of this type, and to sanction their occupation for the purposes to which they are now put. It is not surprising to learn that quite thirty sisters and nurses left the hospital at the beginning of July or August, and it is reasonable to expect that constant changes with increasing difficulty to fill vacancies on the nursing staff must tend to prevail, rather than to diminish, until the existing discomforts and administrative defects are removed.

### Full Knowledge of Faulty Conditions before Engagement.

Dear Sir,—As representing the general feeling of the nursing staff of this hospital regarding the recent criticisms in The Hospital, we would like the public to know that we came here with full knowledge of the conditions under which we should have to live. We were severally warned by the matron, when we applied, that owing to lack of space we should have to sleep in small curtained cubicles, and those who objected need not have come. Still less need they have stayed! Yet, although there have been two opportunities of resigning, the majority of those who came when the building opened fifteen months ago have just recently renewed their contracts, which fact speaks for itself.

We trust that, in common fairness, you will give this protest equal publicity as your statement, which we consider a gross exaggeration and distortion of facts, and a most unwarranted attack on our matron, who has done all in her power from the beginning to promote our interests, and to mitigate any discomfort which the exigency of the situation involved.

Yours truly,

Ward Sisters:—L. Sayer, E. O. Grant, F. F. Orford, A Clarke, F. E. Creese, A. M. Russell, O. Chubb, M. A. Aris, E. M. P. Sketchley, E. M. H. Sparks, K. Marshall, E. M. Cater, A. Blamire, E. M. Carlisle, F. A. Carpenter, A. Coomby, I. L. Riley, J. Thomson, H. B. Hand, M. Cooke, E. Creech, A. E. Hulbert, D. Millson, E. M. Lawson, A. Saserby, Lilian E. Hunting, E. Biglands, F. M. Probert, E. D. Ford, C. Styles, C. Pearce, M. Straston, M. C. Browne, M. Groom.

Staff Nurses:—M. L. Manning, M. Plant, Mary Johnson, E. E. Taylor, S. S. Penning, D. T. Churchman, H. Aubrey, L. G. Collins, E. C. Todhunter, E. Thomas, S. E. Jones, M. E. Taylor, D. M. M. Collett, R. M. Watson, M. A. Munro, K. O'Connell, M. Reid, A. Langmaid, M. Drake, D. W. Frearson, H. A. Macfarlane, E. L. Colnes, D. Edwards, I. M. J. Toughill, O. Browne, H. Chubb, O. Wood, A. Walsh, A. Walker, K. Marsh, E. A. Nullis, N. Tarran, J. Birchall, I. Sturgeon, E. F. Dance, J. Mould, F. Gould, E. Edwards, B. A. B. Smith, A. D. Mackenzie.

*To the Editor,* The Hospital.

King George Hospital, S.E., August 21, 1916.

